# Case Report: Durable Complete Response After Combined Immunotherapy Following Resection of Primary Tumor in a Gallbladder Cancer Patient With Distant Metastatic Lymph Nodes of Favorable Immune-Microenvironment

**DOI:** 10.3389/fimmu.2022.820566

**Published:** 2022-02-15

**Authors:** Bin Yi, Zhikun Zhao, Hui Dong, Lei Yuan, Yingjun Wu, Yun Xu, Xiaoqing Jiang, Chao Sun, Dongfang Wu, Yajie Xiao

**Affiliations:** ^1^ Department of Organ Transplantation, Eastern Hepatobiliary Surgery Hospital, Naval Medical University, Shanghai, China; ^2^ Department of Medicine, YuceBio Technology Co. Ltd., Shenzhen, China; ^3^ Department of Pathology, Eastern Hepatobiliary Surgery Hospital, Naval Medical University, Shanghai, China; ^4^ Department I of Biliary Tract, Eastern Hepatobiliary Surgery Hospital, Naval Medical University, Shanghai, China

**Keywords:** gallbladder carcinoma, chemo-immunotherapy, tumor microenvironment, complete response, distant lymph node metastases

## Abstract

**Background:**

Metastatic gallbladder carcinoma (GBC) is one of the most aggressive malignancies. As GBC is usually diagnosed with distant metastases, only a few patients can receive R0 resection and the relapse rate remains high. Programmed cell death protein 1 (PD-1) blockade therapy has provided encouraging long-term outcomes in a subset of patients in many cancers. However, the data on efficacy of PD-1 blockade in GBC are very limited.

**Case Presentation:**

We herein reported a stage IVB GBC patient with localized primary tumor and distant lymph node metastasis. Except for the unresectable multiple metastatic nodes including distant nodes, a complete resection of primary tumor *en bloc* with partial segment 4B+5 was performed. Tumor tissues of primary tumor and one metastatic lymph node were collected to perform whole-exome sequencing, RNA-seq, and immunohistochemistry. Low TMB (5.38 muts/Mb), low MSI (<20%), and negative PD-L1 expression (TC0) were observed in the primary tumor. Likewise, low TMB (5.44 muts/Mb), low MSI (<20%), and low PD-L1 expression (TC2) presented in the metastatic lymph node. Besides, low genetic intratumor heterogeneity exhibited between the primary and metastatic tumors in this patient. In contrast to the primary tumor, higher-level CD8^+^ T cell infiltration was revealed in the tumor microenvironment of the metastatic lymph node. Then, chemo-immunotherapy using S1 and anti-PD-1 antibody pembrolizumab was administrated as the first-line treatment for the residual metastatic nodes. Complete response was achieved after 7 courses and has lasted for 32 months up to present. Additionally, blood samples during treatment were further analyzed for immune repertoire sequencing, showing that several T cell receptor clones in metastatic lymph node were predominant in blood during the combined anti-PD-1 treatment.

**Conclusions:**

Chemo-immunotherapy may provide a potential curative option for the lymph node metastases of gallbladder cancer. The low intratumor heterogeneity and high level of infiltrating CD8^+^ T-cells in metastatic node might be indispensable to the durable complete response in this patient.

## Background

Gallbladder carcinoma (GBC) is a very aggressive biliary malignancy, accounting for the most common type of biliary tract cancers (BTCs) (1). The incidence rate of GBC is higher in women and elders (2, 3). Epidemiologic studies show that the risk factors of GBC include cholelithiasis, inflammatory bowel disease, diabetes, genetic susceptibility, drinking, and smoking (3). Radical surgery remains the only potentially curable treatment of GBC. Due to lack of specific symptoms and frequent metastases to the liver, lymph nodes, and/or peritoneum, early diagnosis remains difficult and the overall prognosis of advanced diseases is still very poor (2, 3).

Standard first-line chemotherapy has been reported to have limited objective response rates in BTCs; more effective and durable anticancer treatments are needed. To date, increasing applications of immune checkpoint inhibitors (ICIs) in BTCs have been reported (4, 5). Recently, pembrolizumab has shown beneficial effects and good safety for advanced BTC patients in the basket KEYNOTE-158 study and KEYNOTE-028 study, leading to NCCN recommendations for monotherapy in advanced BTC patients of microsatellite instability (MSI)-H status (6). However, it was worth noting that immunotherapy itself might be so far active in a few responders with specific biomarkers such as PD-L1, MSI, tumor mutation burden (TMB), DNA damage repair (DDR), and other predictors (6). Although MSI status is a useful predictive biomarker in addition to PD-L1 expression and TMB, the proportion of BTC patients with MSI-H status was undefined (7). In addition, some infiltrating lymphocytes in the tumor microenvironment (TME) are emerging as predictive or prognostic biomarkers in current anti-cancer immunotherapy (8). A recent *in vitro* study showed that stimulated CD8+ T cells can recognize HLA-matched GBC cell lines, activate DCs, and subsequently present neoantigens (9). Additionally, higher PD-L1 expression and higher CD8+ T cell density together with a good response rate to immunotherapy have been observed in immunologically “hot” BTCs (10).

Therefore, some researchers start to focus on combination strategies with ICIs for advanced BTC (4, 5). For example, an observational study showed prolonged progression-free survival (PFS) in Japanese patients with BTC treating with chemo-immunotherapy than immunotherapy alone (11). More clinical trials for chemo-immunotherapy in advanced BTC are still ongoing (12). Herein, we report a GBC case of distant lymphatic metastases, in which the patient completely responded to first-line chemo-immunotherapy after the complete resection of primary tumor.

## Case Presentation

**Figure 1:**
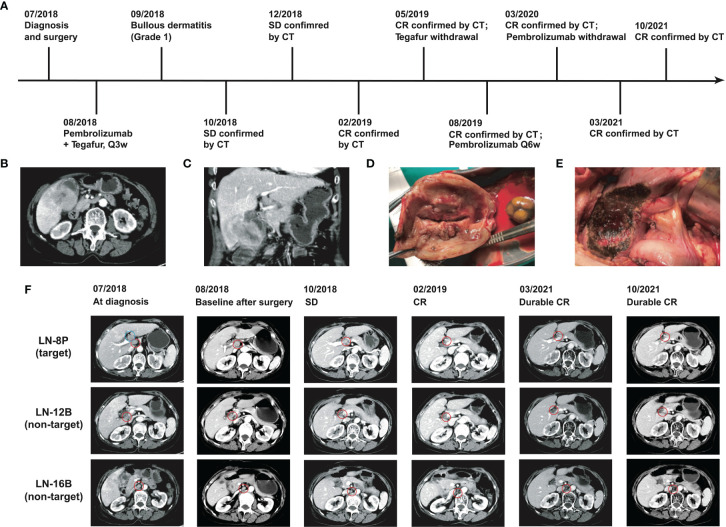
Clinical courses and outcomes of this GBC patient. **(A)** Chronological schema of treatments. **(B)** Transverse images of CT scan. **(C)** Coronal images of CT scan. **(D)** Resection of gallbladder and **(E)** resection on partial liver. **(F)** CT images of the lesions during the treatments. Blue circle indicates the dissected lymph node group-8A.

**Figure 2:**
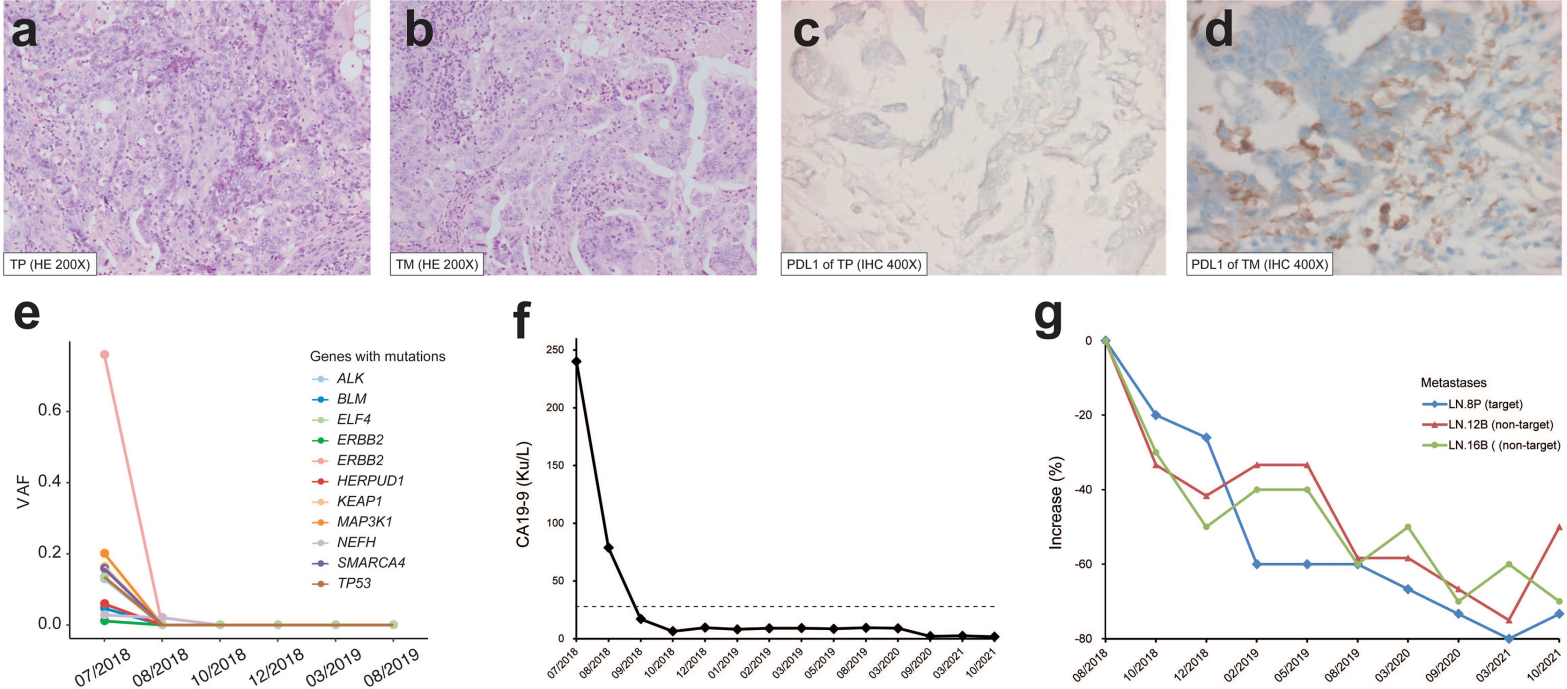
Pathological features and therapeutic response of the patient. H&E staining (×200) of **(A)** primary tumor (TP) and **(B)** lymph node metastasis (TM). PD-L1 staining (×400) of **(C)** TP and **(D)** TM. **(E)** Variant allele frequency (VAF) change of cfDNA mutations during the treatment. **(F)** CA19-9 level during the treatment. The dashed line indicates the normal upper limit. **(G)** Size change of target and non-target lesions of metastatic lymph node. LN-8P with a short diameter of 1.5 cm after surgery on baseline was used for efficacy evaluation.

A 74-year-old female patient with about 10-year history of gallstone was diagnosed with stage IVB gallbladder carcinoma and onset of cholecystitis in July 2018 (**Figure 1A**). The multiple detector computed tomography (MDCT) revealed a solid mass on the gallbladder and adjacent liver, and enlarged regional as well as distant lymph nodes, which appeared with intense uptake on FDG PET/CT (**Figures 1B, C**). After a multidisciplinary discussion, the patient received *en bloc* resection of gallbladder and partial liver (Segment 4B+5) to reduce tumor load as well as to treat drug-ineffective cholecystitis, and dissection of lymph node Group 8A (LN-8A) to confirm metastasis in July 2018 (**Figures 1D–F**). The excised tumors of primary tumor (TP) and metastatic tumor (TM) were confirmed as adenosquamous carcinoma morphologically and immunohistochemically (**Figures 2A, B**). The PD-L1 expressions were TC0 and TC2 in TP and TM, respectively (**Figures 2C, D**). Whole-exome sequencing (WES) of TP and TM revealed a TMB of 5.38 and 5.44 mut/Mb, respectively. Both lesions were MSI-Low based on the sequencing data. We also detected 11 mutations of cfDNA in the serum sample before surgery (**Figure 2E**).

In August 2018, the patient refused to accept gemcitabine plus cisplatin and then began to follow “S1” (tegafur/gimeracil/oteracil, Lunan Pharmaceutical Group, Linyi, China; 40 mg orally twice daily on days 1–14 of a 21-day cycle) plus pembrolizumab (3 mg/kg every 3 weeks) as the first-line treatment. At the baseline assessment after surgery, lymph node Group 8P was the only measurable disease and two smaller nodes were non-measurable according to RECIST 1.1. Meanwhile, one mutation in cfDNA was detected. Bullous dermatitis (grade 1) on both hands occurred after the first administration of pembrolizumab, then significantly relieved in the subsequent cycles, and no other side effects were observed. After the initial 2 cycles of combined treatment, the serum cancer antigen 19-9 (CA19-9) level decreased from 78.9 u/ml to normal, CT scan revealed stable disease with a 20% decrease (**Figures 2F, G**), and no mutations in cfDNA were detected (**Figure 2E**). After 7 cycles of treatment, complete response (CR) achieved with the sum of diameters lessened by 60% and all enlarged nodes shrank to less than 1 cm in the short axis (**Figure 2F**). The same regimens were given for 3 more cycles by the multidisciplinary team, then pembrolizumab monotherapy was continued for another 3 cycles. Afterward, the monoclonal antibody was given every 6 weeks and finally stopped at 1 year of CR. The patient began to present a low fever at 14 months of CR but with normal liver and thyroid functions, and then 2.5 mg prednisone was administrated per day. At the evaluation at 32 months of CR, the serum CA19-9 was kept normal (**Figure 2F**) and CT showed no evidence of disease.

**Figure 3:**
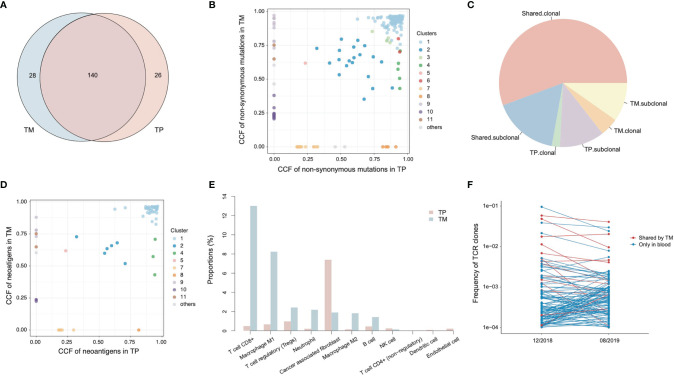
Genetic alterations and tumor microenvironment of primary tumor (TP) and metastatic tumor (TM). **(A)** The Venn diagram of non-synonymous mutations detected in TP and TM. **(B)** The cancer cell fractions (CCF) of non-synonymous mutations detected in TP and TM. **(C)** The percentages of clonality of mutations. **(D)** The CCF of neoantigens calculated in TP and TM. **(E)** The proportions of infiltrating immune cells and stroma cells in TP and TM. **(F)** The frequencies of TCR clones in peripheral blood mononuclear cells (PBMCs).

To investigate the genetic features of the primary tumor (TP) and metastatic lymph node (TM), we first compared the non-synonymous somatic mutations. A total of 140 (71.4%) mutations were shared by the two lesions (**Figure 3A**). In order to clarify the clonality of the mutations, 11 clusters were identified (**Figure 3B**). Cluster 1 was clonal in both TP and TM, accounting for 55.7% of total mutations (**Figure 3C**). In this cluster, several known driver genes were detected, including *TP53*, *ERBB2*, *ELF3*, *PIK3CA*, and *GATA3*. Clusters 2 to 6 were sub-clonal shared mutations. The fractions of private sub-clonal mutations in TP and TM were 11.3% and 9.8%, respectively. The prediction of potential neoantigens was also calculated. Similarly, 75% of neoantigens were shared by TP and TM, and most of the neoantigens were derived from the clonal mutations (**Figure 3D**). No loss of heterogeneity of HLA genes was detected.

To better understand the relationships between primary tumor and metastatic node, we performed RNA-seq to evaluate the tumor microenvironments (**Figure 3E**). In TP, around 8% of all the cells were cancer-associated fibroblasts (CAF), while few infiltrating immune cells were detected. The dominant immune cell type was regulating T cells (T reg) (1%). By contrast, TM showed higher levels of infiltrating CD8+ T cells (13% vs. 0.5%) and M1 macrophages (8% vs. 0.7%) than those in TP, respectively. However, no significant expression differences were observed with any intrinsic genes in the tumor microenvironment. The proportions of T-reg and CAF in TM were 2.4% and 1.9%, respectively. The differences of tumor microenvironments might indicate a suppressed immune state in TP and an activated immune state in TM.

The T cell receptors (TCRs) in primary tumor and metastatic node were also analyzed using RNA-seq data. There was no TCR detected in TP, whereas 99 TCR clones were identified in TM. This was consistent with the abovementioned estimated percentages of T cells. About 77.6% of these TCRs detected in TM appeared as clonal TCRs, revealing the significant expansion of T cells. Two peripheral blood mononuclear cell (PBMC) samples were collected at 2 months of SD and at 6 months of CR during the therapy, for immune repertoire sequencing (IR-seq). A total of 14 TCRs of TM were detected in PBMCs, and 6 of them were shown in top 10 TCRs of PBMCs, indicating a potential association with antitumor function (**Figure 3F**).

## Discussions

Due to the rare prevalence, GBC is often diagnosed at advanced stage and effective treatment options for GBC patients are very limited. Although long-term survival of GBC patients relies on radical surgery, locoregional or distant recurrence frequently happens (13, 14). The current first-line treatment for advanced or metastatic GBC remains chemotherapy with gemcitabine plus cisplatin, while recently the chemotherapy of fluorouracil, folinic acid, and oxaliplatin (FOLFOX) has been recommended as the second-line treatment (13, 15). Nevertheless, other therapeutic options incorporated with targeted therapy or immunotherapy are urgently needed and are being evaluated in many clinical trials for the first-line or subsequent-line use (11, 13, 16).

Herein, we reported a GBC case of localized primary cancer and distant lymphatic metastases, in which durable CR was achieved using anti-PD-1 therapy in combination with “S1” chemotherapy after the resection of primary tumor. In this special case, the metastases occurred only in regional and distant lymph nodes, with no evidence of other extrahepatic metastasis by enhanced CT and PET-CT. As the primary tumor at T stage-3 was still resectable, a complete resection of gallbladder *en bloc* with partial segment 4B+5 was performed in order to remove the infectious gallbladder and to reduce tumor load, and eventually fluoropyrimidine-based chemotherapy combined with PD-1 blockade immunotherapy was adopted for those residual unresectable lymph nodes.

Although PD-L1 expression generally serves as the gold biomarker for immunotherapy, some inconsistent results have emerged due to the variability of immunohistochemical staining antibodies and heterogeneous expressions (17, 18). Furthermore, few studies revealed different PD-L1 positivity rates between matched primary tumors and metastatic lesions. A comprehensive pathological study reported a rare PD-L1 expression in primary prostate cancer but increased rates in metastatic castrate-resistant prostate cancer, consistent with our findings in GBC (19). Therefore, in addition to PD-L1 expression, other biomarkers like TMB are recommended for suitable therapeutic decision making (20). Considering the positive PD-L1 expression (TC2) in TM and intermediate TMB levels in both TM and TP at the time before systemic therapy, we administrated pembrolizumab combined with “S1” for the residual lesions. Interestingly, after the first two cycles of therapy, the target lesion began to shrink, the CA19-9 level dropped to normal, and cfDNA consistently became undetectable. These results justified the therapeutic option and implied a good response to combined immunotherapy. The patient has remained disease-free for 32 months. To our knowledge, this case could be the first of durable CR in stage IV GBC through resection of primary tumor and combined immunotherapy for metastatic disease.

To explore the underlying reasons for the durable CR in this case, we found that a favorable tumor immune-microenvironment in metastatic lymph node could explain the response. Although the data of metastatic LN were derived from resected LN-8A, the data could be the best approach to assess the other metastatic LNs that responded well to the treatment. In this case, low MSI and intermediate TMB shown in both primary tumor and metastatic lesion could hardly be responsible for the durable CR. In addition to PD-L1 expression, other potential factors in the tumor microenvironment are emerging as predictors or prognostic biomarkers in current anticancer therapy (8). Alternatively, we found that the level of intratumor heterogeneity (ITH) for one lesion was extremely low. The ITH values (defined as the fraction of subclonal mutations in all mutations in the exome region of a sample) were 0.264 and 0.234 in TP and TM, respectively. Previous studies have reported that low ITH is associated with better response to immunotherapy and other treatments (21, 22). Hence, the low ITH presented in primary cancer as well as in metastatic node might play an important role in the response to combined immunotherapy. On the other side, we compared the mutation profiling of two lesions. The ITH between primary tumor and metastatic node (defined as 1 - Jaccard similarity index of mutations between TP and TM) was 0.266, also indicating a low ITH between different tumor lesions.

In addition, infiltrating CD8+ T cells in the tumor environment play a key role in neoantigen recognition and in response to immunotherapy (23). Interestingly, we found that the percentage of infiltrating CD8+ T cells was higher in metastatic lymph node than that in primary tumor through RNA-seq. Recently, the levels of tumor-infiltrating lymphocytes were found to be significantly higher in the favorable TME subtype of bladder cancer, which may correlate with clinical response to immunotherapy (24). A recent *in vitro* study showed that stimulated CD8+ T cells can recognize HLA-matched GBC cell lines, activate DCs, and subsequently present neoantigens (9). Another retrospective analysis suggested that high-infiltrating CD8+ T cells density may indicate better OS and PFS in GBC patients (25). Besides, enhanced infiltration of CD4+/CD8+ T cells and dendritic cells were significantly correlated with prolonged survival in patients with resected GBC (26). Additionally, high stromal CD3+ or CD8+ T cells in tumor-infiltrating lymphocytes can predict better survival in GBC patients with small tumors (<3 cm) and immune-based therapy might be beneficial for these patients (27).

Moreover, the TCR clones detected in metastatic lymph node possessed a high frequency in blood and were then sustained during the subsequent treatment. These findings implied that the potential reactivation of CD8+ T cells during anti-PD-1 treatment in unresected metastatic nodes perhaps is important for the durable CR to combined PD-1 blockade. Besides, the infiltrating level of M1 macrophages in the metastatic lymph node was higher than the primary tumor (8% vs. 0.7%), which might also boost the efficacy of immunotherapy (28). Even though the primary tumor of GBC could be classified as an immune “cold” tumor for the immunosuppressive microenvironment (13), the metastatic lymph nodes of GBC within this case or beyond could be in immune “hot” status of a favorable tumor immune-microenvironment and thus might offer a better response to immunotherapy.

In conclusion, we reported a case of stage IVB GBC patients with localized primary tumor and distant lymph node metastasis, in which the patient achieved a durable CR to combined PD-1 blockade after the resection of primary tumor. Low intratumor heterogeneity and high CD8+ cell infiltration might be essential for the response. Chemo-immunotherapy may provide a potential curative option for the lymph node metastases of gallbladder cancer. Expanded cohorts are needed to better clarify the underlying mechanism in this case.

## Data Availability Statement

The datasets presented in this study can be found in online repositories. The whole sequence data reported in this paper available in the Genome Warehouse in National Genomics Data Center, Beijing Institute of Genomics, Chinese Academy of Sciences, under project number PRJCA007420 at https://ngdc.cncb.ac.cn/databases, upon reasonable request.

## Ethics Statement

This study involving human participants was reviewed and approved by the Ethics Committee of Eastern Hepatobiliary Surgery Hospital. The patient provided her written informed consent to participate in this study.

## Author Contributions

BY, XJ, and YJX guided and supervised the project. BY, HD, LY, YW, and YX accomplished the collection and analysis of clinical data. ZZ, CS, DW, and YJX performed the sequencing data analysis. The manuscript was written and revised by ZZ, YJX, and BY. All authors contributed to the article and approved the submitted version.

## Conflict of Interest

Authors ZZ, CS, DW and YJX are employed by YuceBio Technology Co., Ltd.

The remaining authors declare that the research was conducted in the absence of any commercial or financial relationships that could be construed as a potential conflict of interest.

## Publisher’s Note

All claims expressed in this article are solely those of the authors and do not necessarily represent those of their affiliated organizations, or those of the publisher, the editors and the reviewers. Any product that may be evaluated in this article, or claim that may be made by its manufacturer, is not guaranteed or endorsed by the publisher.
